# ASF1B Promotes Oncogenesis in Lung Adenocarcinoma and Other Cancer Types

**DOI:** 10.3389/fonc.2021.731547

**Published:** 2021-09-09

**Authors:** Wencheng Zhang, Zhouyong Gao, Mingxiu Guan, Ning Liu, Fanjie Meng, Guangshun Wang

**Affiliations:** ^1^Department of Oncology, Tianjin Baodi Hospital, Baodi Clinical College of Tianjin Medical University, Tianjin, China; ^2^Department of Thoracic Surgery, Baodi Clinical College of Tianjin Medical University, Tianjin, China; ^3^Department of Laboratory, Tianjin Baodi Hospital, Baodi Clinical College of Tianjin Medical University, Tianjin, China; ^4^Department of Pathology, Tianjin Baodi Hospital, Baodi Clinical College of Tianjin Medical University, Tianjin, China; ^5^Department of Thoracic Surgery, The Second Hospital of Tianjin Medical University, Tianjin, China

**Keywords:** lung adenocarcinoma, immune infiltration cells, prognosis, ASF1B, profiling

## Abstract

Anti-silencing function 1B histone chaperone (ASF1B) is known to be an important modulator of oncogenic processes, yet its role in lung adenocarcinoma (LUAD) remains to be defined. In this study, an integrated assessment of The Cancer Genome Atlas (TCGA) and genotype-tissue expression (GTEx) datasets revealed the overexpression of ASF1B in all analyzed cancer types other than LAML. Genetic, epigenetic, microsatellite instability (MSI), and tumor mutational burden (TMB) analysis showed that ASF1B was regulated by single or multiple factors. Kaplan-Meier survival curves suggested that elevated ASF1B expression was associated with better or worse survival in a cancer type-dependent manner. The CIBERSORT algorithm was used to evaluate immune microenvironment composition, and distinct correlations between ASF1B expression and immune cell infiltration were evident when comparing tumor and normal tissue samples. Gene set enrichment analysis (GSEA) indicated that ASF1B was associated with proliferation- and immunity-related pathways. Knocking down ASF1B impaired the proliferation, affected cell cycle distribution, and induced cell apoptosis in LUAD cell lines. In contrast, ASF1B overexpression had no impact on the malignant characteristics of LUAD cells. At the mechanistic level, ASF1B served as an indirect regulator of DNA Polymerase Epsilon 3, Accessory Subunit (POLE3), CDC28 protein kinase regulatory subunit 1(CKS1B), Dihydrofolate reductase (DHFR), as established through proteomic profiling and Immunoprecipitation-Mass Spectrometry (IP-MS) analyses. Overall, these data suggested that ASF1B serves as a tumor promoter and potential target for cancer therapy and provided us with clues to better understand the importance of ASF1B in many types of cancer.

## Introduction

Lung cancer is one of the most common and deadliest forms of malignant cancer throughout the world (1). Approximately 40% of lung cancer cases are of the most common LUAD histopathological subtype (2). LUAD is associated with high rates of tumor recurrence and a poor prognosis owing to the combination of adverse factors that span a range of different biological and clinical behaviors and the increased resistance to anti-lung cancer drugs. Moreover, existing targeted drugs have shown unsatisfactory efficacy (3). Further research is thus needed to better understand the mechanisms underlying LUAD development and progression. Genetic mutation is the primary process that drives oncogenesis (4, 5), with gene-specific overexpression or silencing being additionally associated with epigenetic mechanisms such as changes in histone post-translational modification or DNA methylation (6–8). Aberrant activation or expression of chromatin-regulating proteins such as histone-modifying enzymes, histone variants, effector proteins, histone chaperones, and chromatin remodeling proteins is closely tied to cancer onset and progression (9–11). Histone H3–H4 chaperone anti-silencing function 1 (ASF1) is a key histone chaperone involved in regulating processes including DNA replication, DNA damage repair, and transcription (12, 13). There are two paralogous forms of ASF1: Anti-Silencing Function 1A Histone Chaperone (ASF1A) and ASF1B. While ASF1A is primarily involved in regulating DNA repair and cellular senescence, ASF1B serves as a preferential regulator of cellular proliferation (13, 14). Increased ASF1B expression levels have been linked to the prognosis of LUAD and breast cancer patients (15, 16). Prior work suggests that ASF1B is a key regulator of proliferation, apoptosis, and the cell cycle in prostate cancer, cervical cancer, clear cell renal cell carcinoma, and breast cancer (16–19). Even so, the role of this gene in LUAD and many other cancers has yet to be definitively established. Herein, we explored the expression and prognostic relevance of ASF1B across cancers, in addition to evaluating the association between ASF1B expression levels and molecular pathways, immune infiltration, methylation, Copy number variations (CNV), MSI, and TMB. Lastly, we examined the impact of knocking down and overexpressing ASF1B on proliferation, cell cycle progression, apoptosis, and potential mechanism of LUAD. Our data provide novel insights into the functional importance of ASF1B in LUAD and indicate ASF1B as a potential target for the therapeutic management of cancers.

## Materials and Methods

### Dataset Analyses

To evaluate the expression of ASF1B in 33 different cancers, TCGA was queried to download RNA-seq gene expression data and clinical records pertaining to 11,058 cases (http://xena.ucsc.edu/welcome-to-ucsc-xena/) (Workflow Type: HTSeq FPKM) (20), with the GTEx data similarly being downloaded (21). Samples for which data pertaining to age, gender, TNM stage, distant and lymph node metastases, or OS were not recorded were excluded from subsequent analyses, as were patient samples with an Overall Survival (OS) <30 days. GSE31210 and GSE62254 datasets was downloaded from GENE EXPRESSION OMNIBUS (GEO). ICGC_ARRAY dataset was downloaded from International Cancer Genome Consortium (ICGC). Levels of ASF1B expression in Pan-cancer and normal tissue datasets were additionally evaluated with the Oncomine database (http://www.oncomine.org) (22–48). Relationships between methylation and patient outcomes were assessed with the MethSurv database (https://biit.cs.ut.ee/methsurv/) (49). Associations between ASF1B expression, methylation, and CNVs were examined using the GSCALite platform (http://bioinfo.life.hust.edu.cn/web/GSCALite/) (50). Correlations between ASF1B and molecular-or immune-related subtypes were assessed with the TISIDB platform (TISIDB (hku.hk)) (51).

### Immune Infiltration Analysis

The CIBERSORT algorithm was used to approximate the infiltration of different immune cell types into patient tumors, followed by quality filtering. Additionally, the R ESTIMATE algorithm was utilized to assess tumor purity for all samples (52).

### Cell Culture and Transfection

H1299, H1975, H1650 cells were grown in RPMI-1640 (Gibco BRL, MD, USA), while A549 cells were cultured in DMEM (Gibco BRL). In both cases, media contained 10% fetal bovine serum (Gibco BRL), 50 IU/mL penicillin, and 50 mg/mL streptomycin (Invitrogen, CA, USA), and cells were grown in humidified 5% CO_2_ incubators at 37°C. Three different ASF1B-specific small-interfering RNA (siRNA) constructs were synthesized (siASF1B-1: CAACGAGUACCUCA ACCCUTT, siASF1B-2: GACGACCUGGAGUGGAAUUTT, siASF1B-3: UCAACUGCACUC CUAUCAATT. GenePharma, Shanghai, China), another siRNA derives from the literature (siASF1B-4: CCCUUGAGUACCAUUGAUCUU) (53). They were transiently transfected into cells using Lipofectamine 2000 (Invitrogen) based on provided directions. At 60 h post-transfection, Western blotting was used to select the most effective siRNA. Next, short hairpin RNAs (shRNA) constructs were synthesized based upon the most effective siRNA sequence (Gene Pharma). ASF1B overexpression and negative control lentiviral factors were obtained from FENGHUISHENGWU (Changsha, Hu Nan Province, China), while lentiviruses encoding ASF1B-shRNA and corresponding negative controls were from Gene Pharma. LUAD cells were transduced with these lentiviral vectors at stock concentrations of 1×10^8^ –1×10^9^ particles/ml. The H1975 and H1650 cell lines were used for gain-of-function studies, whereas H1299 and A549 cells were utilized when conducting loss-of-function studies. Cellular transduction was performed when cells were 60–70% confluent, with lentiviruses being administered at a dose of 1×10^7^/ml together with 6 μg/ml polybrene (Sigma-Aldrich, H9268). After transduction, puromycin (0.6 µg/mL, Sigma) was used to select for stably transduced cells.

### EdU Assay

Cells were incubated for 2 h with EdU (Ribobio, Guangzhou, China), after which they were process sed based on provided directions. Cells were washed thrice with phosphate buffered saline (PBS), treated for 50 min with 100μl of 1×Apollo^®^ reaction cocktail, and stained for 30 min with 100 μl of Hoechst 33342 prior to fluorescent microscopic visualization.

### CCK-8 Assay

Cell viability was assessed *via* CCK-8 assay (gene-protein link, Beijing, China) Briefly, cells were added to 96-well plates (2x10^3^/well) for 24, 48, 72, or 96 h, after which 10 µl of CCK-8 solution was added per well and plates were incubated for an additional 2 h at 37°C. Absorbance at 450 nm was then assessed with an iMark microplate reader (Bio-Rad), with six wells per treatment group being analyzed.

### Flow Cytometry

Cell cycle progression was assessed by fixing cells overnight with chilled 70% ethanol at 4°C. Cells were then washed in PBS and suspended in 415 ul of propidium iodide (PI, gene-protein link) for 30 min at 37°C while protected from light. A flow cytometer (BD Accuri C6, BD Biosciences, USA) was then used to assess the cells, with the resultant data being analyzed using ModFit LT. Cellular apoptosis was assessed with an Annexin V-AF647/PI kit (Gene-Protein Link) based on provided directions. Briefly, cells were washed twice with chilled PBS, resuspended in binding buffer, and 2x10^5^ cells in 100 ul were stained with 5µL of Annexin V-AF647. Samples were gently mixed for 5 min at room temperature, after which 10 uL of PI was added. Finally, 400 uL of PBS was added, and samples were assessed *via* flow cytometry.

### Western Blotting

Nuclear proteins were isolated from cells with the NE-PER™ Nuclear and Cytoplasmic Extraction Reagents (Thermo Fisher), and a NanoDrop ONE instrument was used to quantify protein levels in each sample. Protein from ~1x10^6^ appropriately treated cells was then extracted with 1x SDS loading buffer, and Western blotting was conducted as described previously (54). Briefly, equal protein amounts were separated *via* 10% or 15% SDS-PAGE and transferred onto PVDF membranes (0.22u m, Bio-rad), which were blocked for 1 h using 5% non-fat dry milk in Tris buffered saline (TBS) containing 0.05% tween-20 (TBST) (Solarbio Life Sciences, Beijing, China) at room temperature, followed by overnight incubation at 4°C with appropriate primary antibodies. Blots were then stained with secondary antibody (1:5000, ZSGB-BIO, ZB-2305, ZB-2301) for 1 h at room temperature, and ECL reagents (CWBIO) were used to detect protein bands. Anti-ASF1B was purchased from Santa Cruz Biotechnology (1:200, sc-393169), while anti-POLE3 was from Proteintech (1:2000, 15278-1-AP), and anti-CASP-3 was from Cell Signaling (1:1000, 9662).

### Immunofluorescent Staining

H1975 Cells were added to glass coverslips in 6-well plates, fixed with 4% formaldehyde, permeabilized with 0.5% Triton X-100/PBS, blocked for 30 min with 5% Albumin Bovine V (BSA) at room temperature, and incubated overnight with anti-ASF1B (1:100, Santa Cruz Biotechnology, Inc. sc-393169.) at 4°C. Cells were then probed with a secondary fluorescently conjugated antibody (1:300,bs-0295G) for 2 h, followed by DAPI counterstaining (Solarbio Life Science), after which images were captured *via* inverted fluorescence microscopy (Nikon, Japan).

### Proteomic Profiling

Proteomic analyses were performed as in prior reports (55). Briefly, following protein isolation and trypsin treatment, peptides were dissolved in water containing 0.1% formic acid in water and analyzed *via* liquid chromatography-tandem mass spectrometry (LC-MS/MS). Raw MS data were converted into a generic Mascot file using Proteome Discoverer (Thermo Scientific, v 2.0), and were processed with the Mascot search engine (Matrix Science, v.2.3.02).

### IP-MS

Nuclear proteins were extracted from control and ASF1B-3x Flag-expressing A549 cells using the NE-PER™ Nuclear and Cytoplasmic Extraction Reagents to which protease and phosphatase inhibitors had been added. Supernatants were mixed for 2 h with anti-Flag at 4°C, after which they were mixed for 1 h with A/G agarose beads (Thermo Fisher Scientific) at 4°C. Protein complexes were then rinsed four times using NETN, one time with PBS, and separated *via* 10% SDS-PAGE. Coomassie blue was used to stain gels in order to visualize proteins, with gel lanes then being excised for in-gel tryptic digestion. Peptides were then extracted, concentrated, and analyzed *via* LC-MS/MS (EASY nLC 1200-Orbitrap Fusion Lumors+ETD, Thermo Fisher Scientific).

### qRT-PCR

TRIzol (Invitrogen) was used to extract RNA from appropriate cells based on provided directions, after which SuperScript III First-strand (Thermo Fisher) was used to prepare cDNA. Primers used in Quantitative Real-time PCR (qPCR) assays are shown in **Table S1**. All qPCR reactions were conducted with SYBR Green Master Mix (Thermo Fisher) using the following conditions: 95°C for 5min; 40 cycles of 95°C for 30 s and 60°C for 1 min. β-actin served as a normalization control.

### Statistical Analysis

Data were analyzed using R v 3.6.3. Wilcoxon tests were used to compare ASF1B expression levels in normal and tumor tissues, while Kruskal-Wallis tests were used to evaluate relationships between ASF1B expression and patient clinical stage. Kaplan-Meier curves were used to assess survival outcomes, and correlations were evaluated with Spearman’s correlation coefficients. A two-sided P < 0.05 was the threshold of significance.

## Results

### Assessment of ASF1B Expression in Cancer

**Figure 1:**
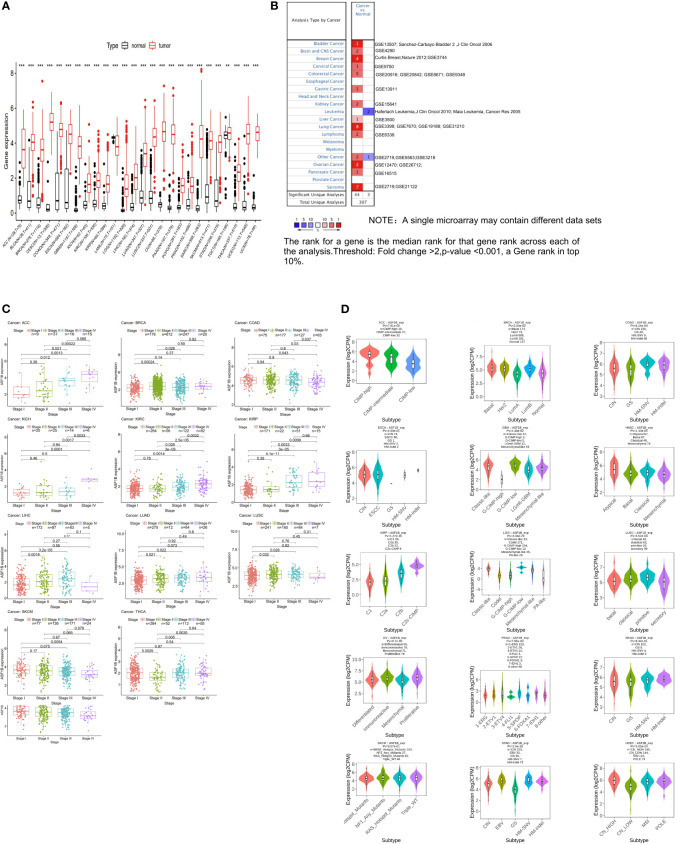
The transcription levels of ASF1B in human cancers. **(A)** The mRNA expression of ASF1B between tumor and normal tissues was analyzed by using tissues from TCGA and GTEx. **(B)** The mRNA expression of ASF1B between tumor and normal tissues was analyzed by using tissues from Oncomine. **(C)** Correlations of ASF1B expression with different clinical stages in patients with different cancers from TCGA. **(D)** ASF1B expression in different molecular subtypes of cancers *via* TISIDB database (**p value ≤ 0.01; ***p value ≤ 0.001).

We began by querying the GTEx and TCGA databases, revealing pronounced ASF1B upregulation in all cancers other than Acute Myeloid Leukemia (LAML) (**Figure 1A**), as further confirmed using Oncomine data (**Figure 1B**). We also found that ASF1B expression levels varied significantly among different clinical stages in patients with Adrenocortical carcinoma (ACC), Breast invasive carcinoma (BRCA), Colon adenocarcinoma (COAD), Kidney Chromophobe (KICH), Kidney renal clear cell carcinoma (KIRC), Kidney renal papillary cell carcinoma (KIRP), Liver hepatocellular carcinoma (LIHC), LUAD, Lung squamous cell carcinoma (LUSC) and Skin Cutaneous Melanoma (SKCM) (**Figure 1C**). In addition, ASF1B expression in different molecular subtypes of ACC, BRCA, COAD, Esophageal carcinoma (ESCA), Glioblastoma multiforme (GBM), Head and Neck squamous cell carcinoma (HNSC), KIRP, Brain Lower Grade Glioma (LGG), LUSC, Ovarian serous cystadenocarcinoma (OV), Prostate adenocarcinoma (PRAD), Rectum adenocarcinoma (READ), SKCM, Stomach adenocarcinoma (STAD), Uterine Corpus Endometrial Carcinoma (UCEC) was significantly different (**Figure 1D**).

### Evaluation of the Prognostic Relevance of ASF1B in Different Cancers

**Figure 2:**
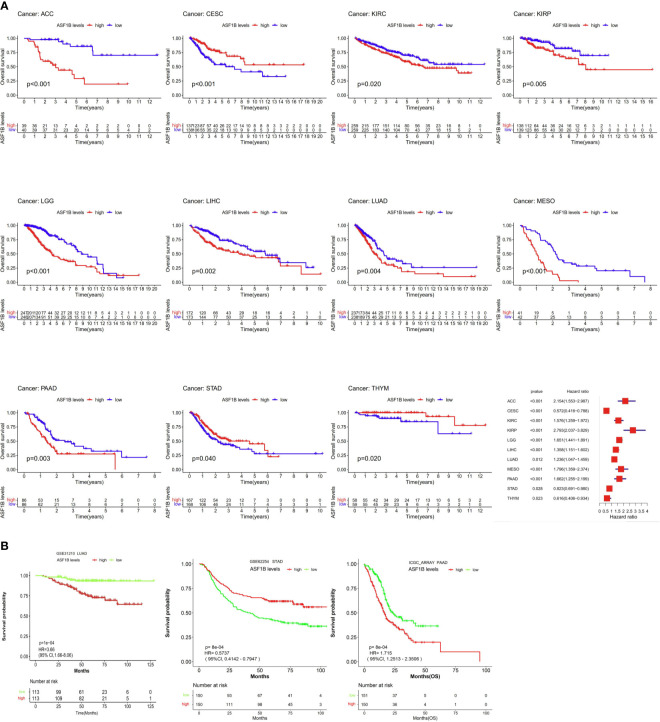
The association between ASF1B expression and cancer patient prognosis. **(A)** The correlation between ASF1B expression and the prognosis of various cancer types were evaluated by The TCGA database. **(B)** The Relationship between ASF1B expression and the prognosis of various cancer types were analyzed.

Next, we examined the prognostic relevance of ASF1B in different cancer types in order to determine whether it was consistently associated with particular cancer patient outcomes. Elevated ASF1B expression was linked to poorer OS in ACC, KIRC, KIRP, LGG, LIHC, LUAD, Mesothelioma (MESO), and Pancreatic adenocarcinoma (PAAD), whereas it was associated with better OS in Cervical squamous cell carcinoma and endocervical adenocarcinoma (CESC), STAD, and Thymoma (THYM) patients (**Figure 2A**). Subsequent GSE31210, GSE62254 and ICGC_ARRAY datasets analysis supported results of LUAD、PAAD and STAD (**Figure 2B**).

### Assessment of the Association Between ASF1B Expression and Methylation, MSI, TMB, and Genetic Alteration Status in Different Cancers

**Figure 3:**
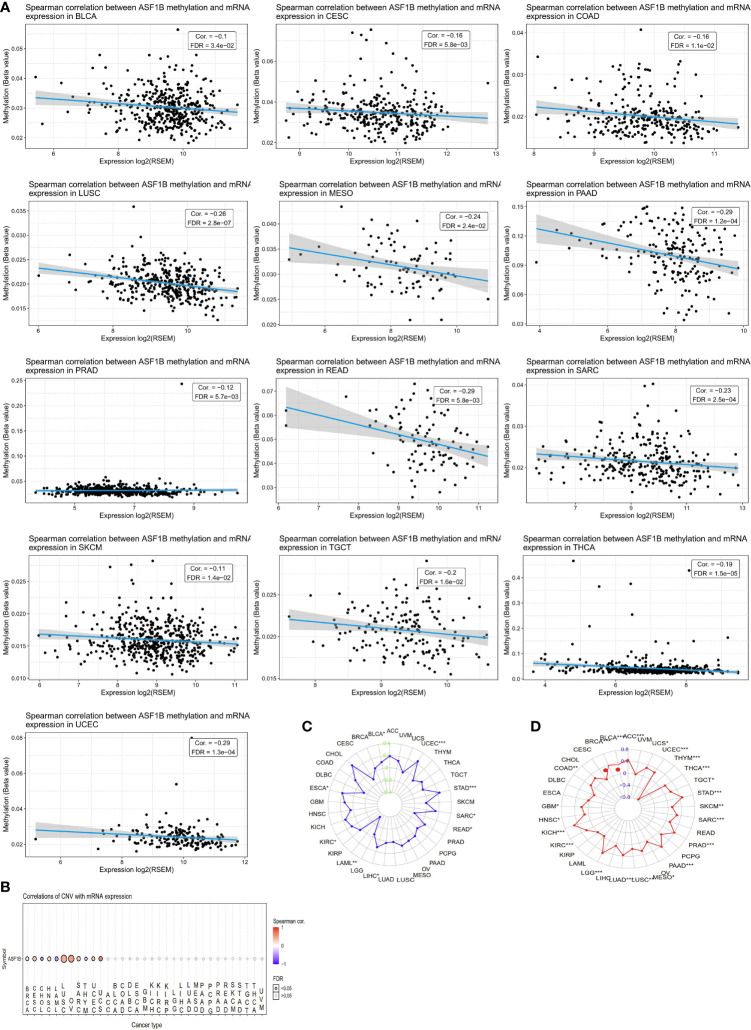
CNV, DNA methylation, MSI and TMB of ASF1B in human cancers. **(A)** The relationship between methylation and ASF1B expression. DNA methylation beta values ranging from 0(unmethylated) to 1(fully methylated). **(B)** Correlations of CNV and ASF1B expression. **(C)** Correlations of TMB and ASF1B expression. **(D)** Correlations of MSI and ASF1B expression. *p value ≤0.05; **p≤0.01; *** p value ≤0.001.

We next sought to explore whether ASF1B expression patterns and prognostic relevance were related to patterns of DNA methylation in different cancer types. A negative association between ASF1B expression levels and DNA methylation were observed in ACC, Bladder Urothelial Carcinoma (BLCA), CESC, COAD, LGG, LIHC, LUSC, MESO, PAAD, READ, PRAD, Sarcoma (SARC), SKCM, STAD, Testicular Germ Cell Tumors (TGCT), Thyroid carcinoma (THCA), and UCEC (P<0.05) (**Figure 3A**). When we further evaluated methylated sites associated with prognostic outcomes in different cancers, we found that cg2527424, cg26259181, cg25274248, cg06391548, and cg26259181 were related to poorer survival in KIRP, LGG, LIHC, STAD, and LUAD (P<0.05), respectively (**Supplementary Figure 1**). To assess the degree of variability in ASF1B expression attributable to CNVs we additionally conducted correlation analyses revealing a positive association between ASF1B expression and CNVs in BRCA, CESC, HNSC, LUSC, OV, SARC, UCEC, and Uterine Carcinosarcoma (UCS), whereas this correlation was negative in ACC, LAML, and THYM (P<0.05) (**Figure 3B**). MSI referred to the spontaneous loss or gain of nucleotides from short tandem repeat DNA tracts (56), and we thus examined correlations between ASF1B expression and MSI status, indicating a positive association between these variables in BLCA, UCEC, STAD, SARC, LIHC, KIRC, and ESCA, whereas they were negatively correlated in READ, and LAML (P<0.05) (**Figure 3C**). TMB is emerging as a profound biomarker for predicting immunotherapy effect and is calculated as total amount of mutations per DNA megabases, in which the detected variants are defined as insertions, base substitutions, or deletions across bases (57). We also assessed the relationship between TMB and ASF1B expression, revealing them to be positively correlated in ACC, BLCA, BRCA, COAD, GBM, HNSC, KICH, KIRC, LGG, LUAD, LUSC, MESO, PAAD, PRAD, SARC, STAD, TGCT, THCA, UCEC, and UCS, but negatively correlated with THYM (P<0.05) (**Figure 3D**). As such, aberrant ASF1B expression and associated prognostic relevance in different cancers may be partially attributable to the above mechanisms.

### The Association Between ASF1B Expression and Immune Cell Infiltration

**Figure 4:**
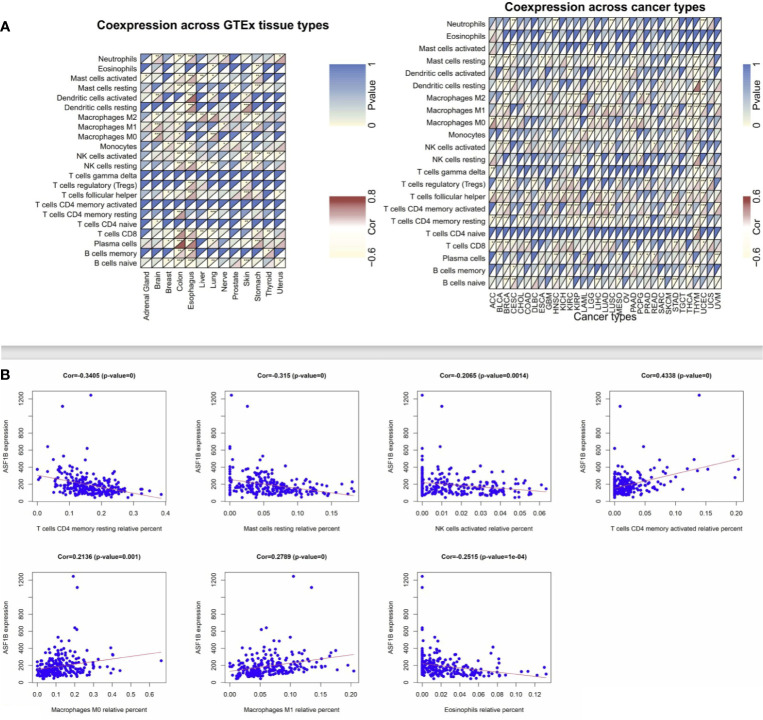
Correlation analysis between ASF1B and tumor-infiltrating immune cell. **(A)** Correlation analysis of ASF1B mRNA expression with 22 types of immune cells were explored across cancers and normal tissues from TCGA and GTEx by CIBERSORT. **(B)** Correlation analysis of ASF1B mRNA expression with immune cells were further investigated in LUAD from GEO by CIBERSORT. *p value ≤0.05; **p≤0.01; *** p value ≤0.001.

Next, we employed the CIBERSORT algorithm to assess relationships between immune cell infiltration and ASF1B expression in tumor and normal tissue samples. GEO and TCGA results revealed ASF1B expression to be positively correlated with levels of M1 and M0 macrophages as well as with levels of activated memory CD4+ T cells, whereas it was negatively correlated with resting memory CD4+ T cells and resting Mast cells in LUAD (**Figures 4A, B**). In lung tissue samples from the GTEx database, ASF1B expression was positively correlated with resting memory CD4+ T cells and negatively correlated with M0 macrophages. When we expanded these results to other tumors and normal tissue types, we found ASF1B to be unrelated to gamma delta T cell or activated memory CD4+ T cell infiltration in normal tissues, and it was similarly unrelated to naïve CD4+ T cell infiltration in analyzed cancers. ASF1B was associated with M2 macrophages in 7 cancers, resting Mast cells and activated NK cells in 6 cancers, M0 and M1 macrophages in 7 cancers, T follicular helper cells in 11 cancers, and resting memory CD4+ T cells in 10 cancers. ASF1B was also associated with resting Mast cells, neutrophils, activated Mast cells, B cells, CD8+ T cells, and naïve CD8+ T cells in 5 normal tissues, M0 macrophages in 3 normal tissues, plasma cells and M1 macrophages in 4 normal tissues, and activated NK cells and M2 macrophages in 6 normal tissues (**Figure 4A**). Through molecular immune subtyping, we further observe significant differences in ASF1B expression levels across C1(wound healing), C1(IFN-γ dominant), C3(inflammatory), C4 (lymphocyte deplete), C5(immunologically quiet), and C6 (TGF-β dominant) subtypes for most analyzed cancers (**Supplementary Figure 2**).

### Examination of Pathways Significantly Associated With ASF1B

**Figure 5:**
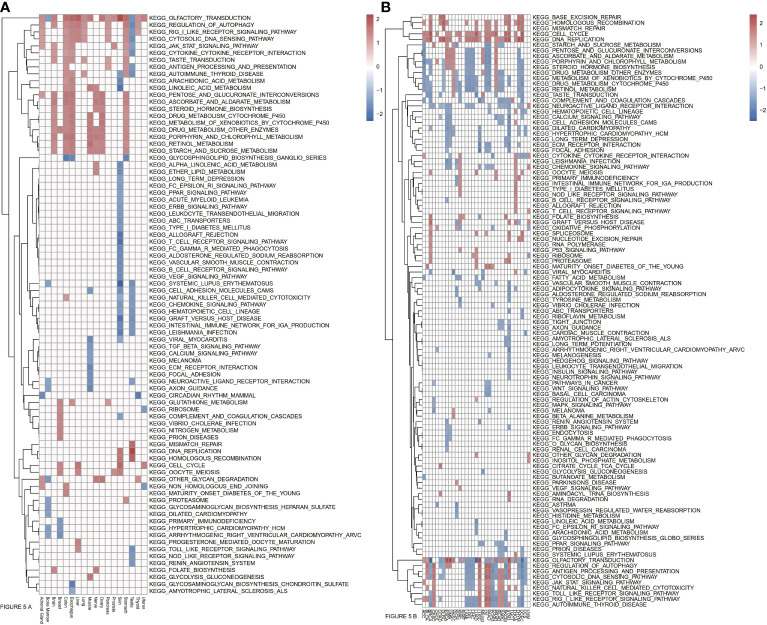
**(A)** Relationships between ASF1B and KEEG pathways in normal tissues from GTEx analyzed by GSEA. **(B)** Relationships between ASF1B and KEEG pathways in cancers from TCGA analyzed by GSEA. (NES≥1.0, p-value<0.05).

To more fully explore the functional roles of ASF1B, we conducted a KEGG GSEA assessment across tumor and normal tissue types, with the resultant heatmap exhibiting a clear clustering pattern (P<0.05, NES>1, NES<-1). Immune-related pathways were highly enriched in normal tissues, with ASF1B being significantly related to JAK/STAT signaling in 7 normal tissues, cytosolic DNA sensing and RIG-I-like receptor signaling in 9 normal tissues, cytokine-cytokine receptor interactions in 5 normal tissues, antigen processing and presentation in 4 normal tissues, autophagy regulation in 10 normal tissues, and the cell cycle and oocyte meiosis in 4 normal tissues (**Figure 5A**). In pan-cancer analyses, ASF1B was significantly associated with Toll-like receptor signaling in 11 cancers, NK cell-mediated cytotoxicity in 14 cancers, chemokine signaling in 10 cancers, JAK/STAT signaling in 3 cancers, Cytosolic DNA sensing in 20 cancers, cytokine-cytokine receptor interactions in 9 cancers, antigen processing and presentation in 19 cancers, autophagy regulation in 20 cancers, the cell cycle in 21 cancers, cell adhesion molecules in 6 cancers, DNA replication in 22 cancers, vascular smooth muscle contraction in 4 cancers, homologous recombination in 15 cancers, mismatch repair in 8 cancers, and ECM receptor interaction in 8 cancers. We further identified four pathways that were only evident in different cancers, with ASF1B being significantly involved in the regulation of base excision repair in 7 cancers, pathways in cancer in 3 cancers, P53 signaling pathway in 3 cancers, and the spliceosome in 5 cancers (**Figure 5B**).

### ASF1B Regulates Lung Cancer Cell Line Phenotypes

**Figure 6:**
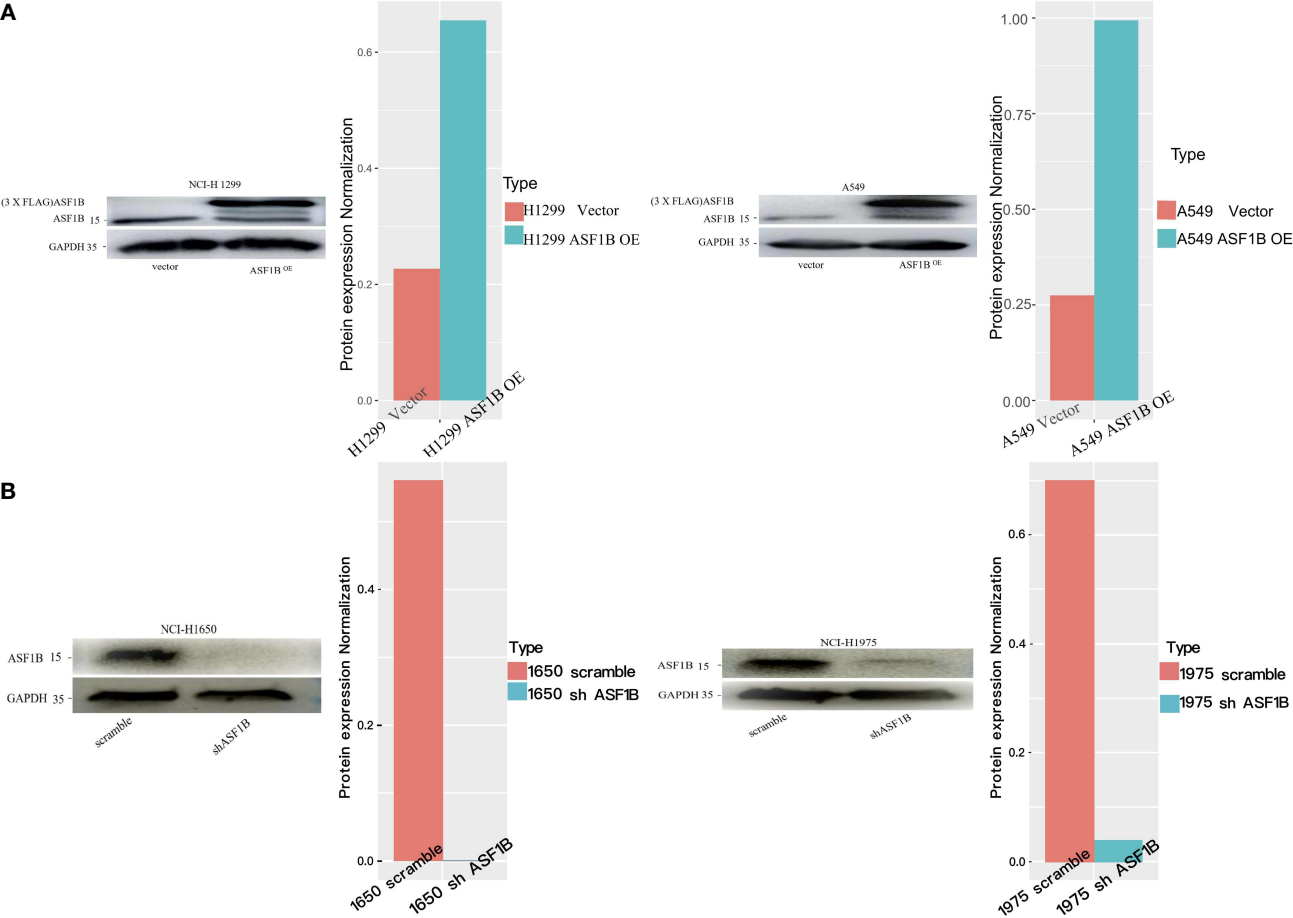
Knockdown or overexpression of ASF1B in LUAD cell lines. **(A)** ASF1B protein expression in stable ASF1B-OE cells. Western blot detecting higher ASF1B protein levels in stable ASF1B-OE-H1299 or ASF1B-OE-A549 cells than those in control cells. The density levels were quantified and represented as a bar graph. **(B)** ASF1B protein expression in stable ASF1B-shRNA-cells. Western blot detecting lower ASF1B protein levels in stable ASF1B-shRNA-H1975 or ASF1B-shRNA-H1650 cells than those in control cells.

Next, we measured ASF1B expression levels in different LUAD cell lines (A549, NCI-H1975, NCI-H1299, NCI-H1650) using data from the Cancer Cell Line Encyclopedia (CCLE) database, revealing that these levels ranged from low (A549 cells) to very high (H1650 cells) (**Supplementary Figure 3**). To understand the functional role of this gene in LUAD, we knocked it down in H1975 and H1650 cells and overexpressed it in two other cell lines. The efficiency of ASF1B knockdown was assessed using four different siRNA constructs, with subsequent Western blotting revealing siRNA-4 to be the most effective in H1975 cells (**Supplementary Figure 4**). Lentiviral vectors were then used to generate stable cell lines (**Figure 6**).

**Figure 7:**
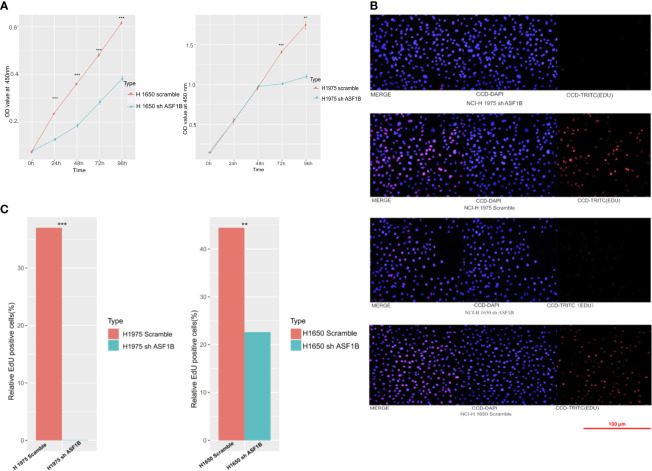
**(A)** Cell viability assay of H1975 and H1650 cells. **(B)** Cell proliferation assay of H1975 and H1650 cells. **(C)** Accumulated analysis of the cell proliferation. **p vaule ≤0.01; ***p vaule ≤0.001.

**Figure 8:**
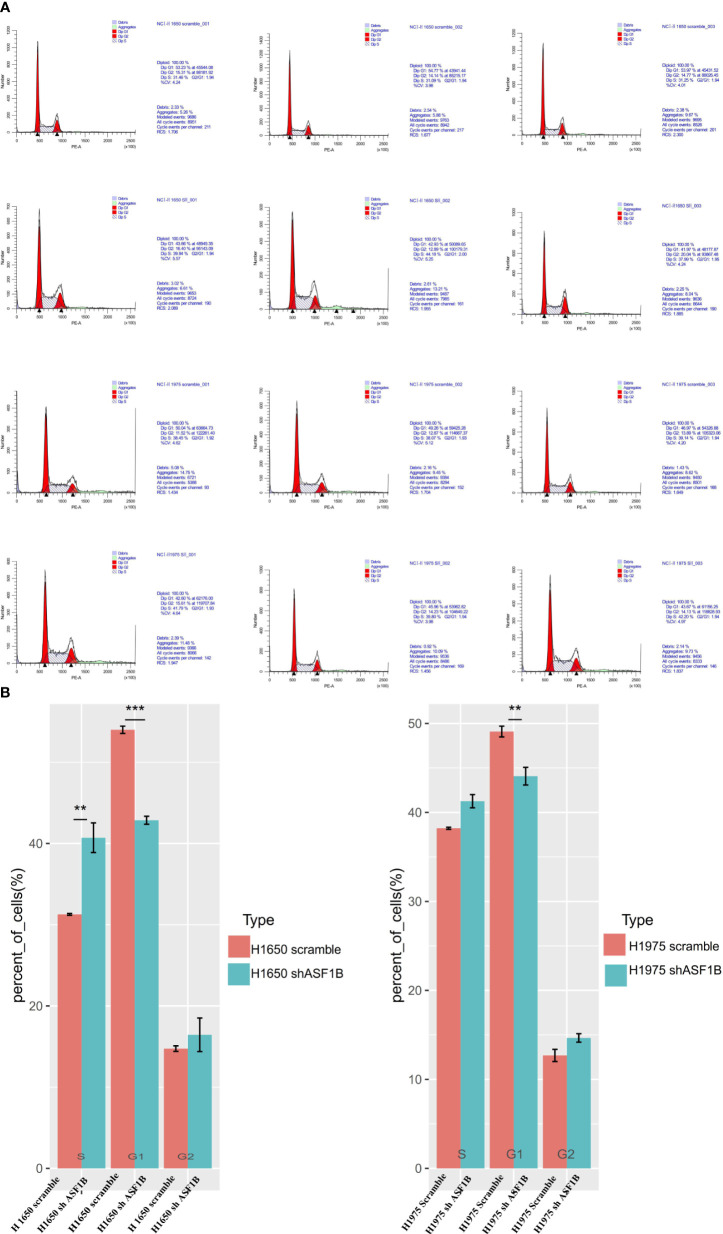
ASF1B knockdown influence the cell cycle. **(A)** Flow cytometry was used to detect the cell cycle changes in H1975 and H1650. **(B)** Accumulated analysis of the cell cycle. **p vaule ≤0.01; ***p vaule ≤0.001.

In CCK-8 assays, ASF1B knockdown markedly impaired H1975 and H1650 cell viability relative to scramble controls (P<0.05) (**Figure 7A**). Consistently, in an EdU uptake assay these ASF1B-knockdown cells exhibited impaired proliferation (P<0.05) (**Figures 7B, C**). Flow cytometric analyses additionally indicated that such knockdown was associated with a significant increase in the percentage of cells in the S phase only in H1650 cells (P<0.05) and with a significant reduction in the frequency of cells in the G1 phase (P<0.05) relative to scramble control in H1650 and H1975 cells (**Figure 8**).

**Figure 9:**
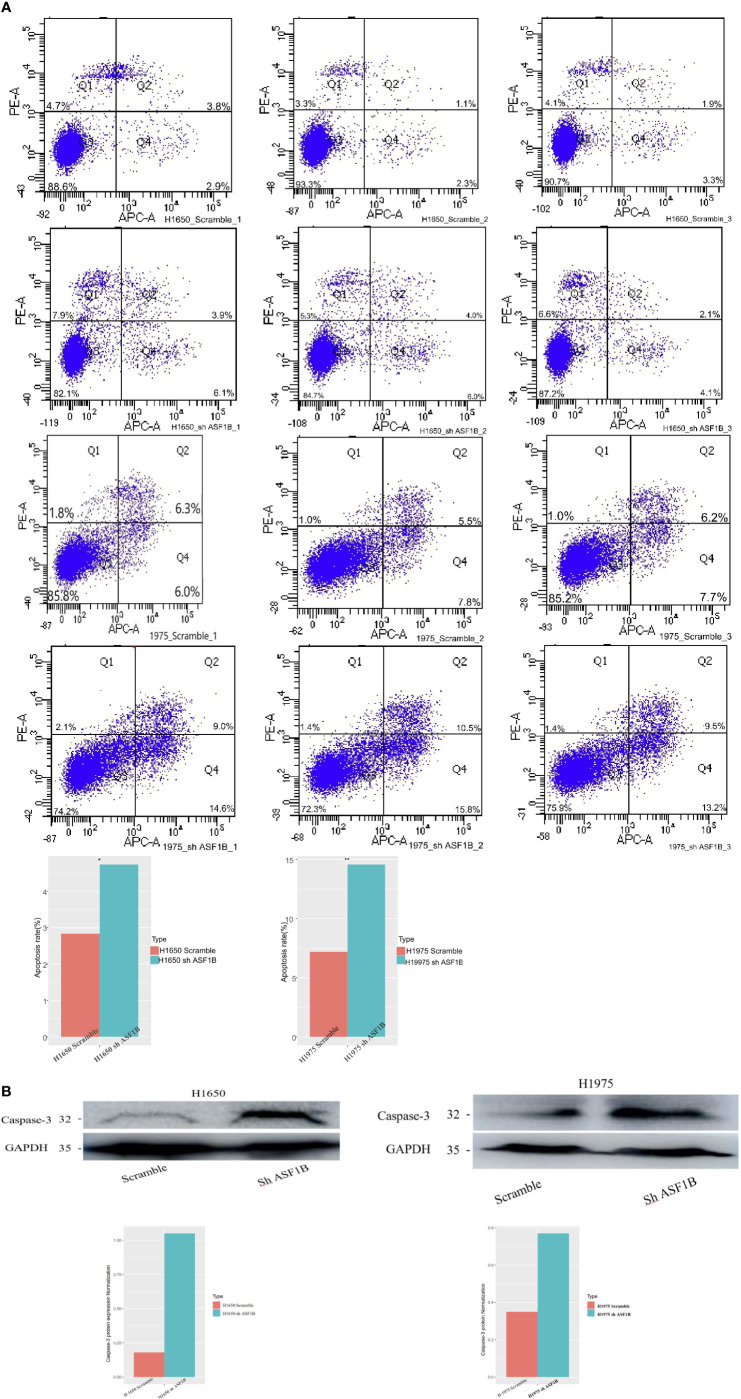
**(A)** Flow cytometry was used to detect the early apoptosis changes in H1975 and H1650. **(B)** Western blot was used to detect the protein levels of apoptosis-3 in H1650 and H1975 cells treated with knockout-ASF1B and untreated control cells. *p vaule ≤0.05; **p vaule ≤0.01.

In addition, ASF1B knockdown was linked to an increase in apoptotic cell death as measured *via* flow cytometry (**Figure 9A**). To confirm that ASF1B is associated with apoptosis in LUAD cells, we analyzed caspase-3 levels therein, revealing a significant increase in caspase-3 levels in H1975 and H1650 cells following ASF1B knockdown (**Figure 9B**). Overall, these findings indicated that ASF1B downregulation can inhibit proliferation, modulate cell cycle progression, and promote apoptosis. In lung cancer cells. ASF1B overexpression did not affect these processes (data not shown).

### Proteomic Profiling-Based Identification of ASF1B Downstream Signaling Target Proteins

**Figure 10:**
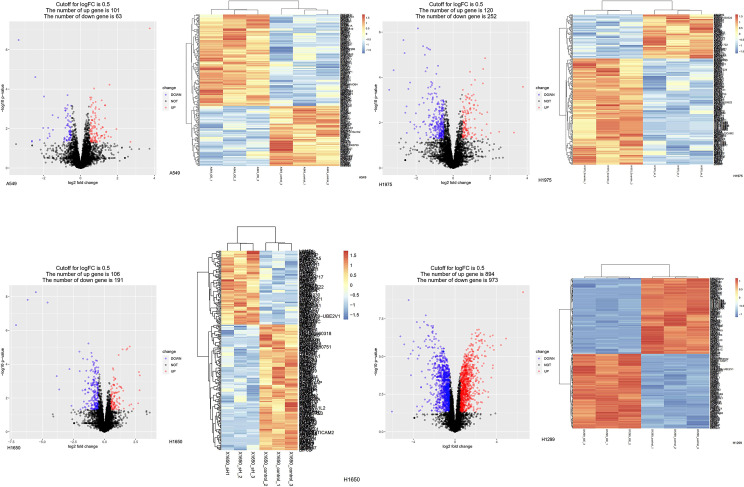
Protein changes of LUAD cell lines induced by knockdown or overexpressed of ASF1B. Differentially expressed proteins in stable transfection cell lines compared to negative control. (Volcano plot) Red presents up-regulated proteins, blue represents down-regulated proteins, and black presents no significantly differentially expressed proteins. (Heatmap) The expression patterns of these differentially expressed proteins can distinguish between stable transfection cell lines and negative control.

To explore the downstream mechanisms whereby ASF1B may influence the above pathways, an LC-MS analysis was conducted to screen for ASF1B target proteins in four cell lines (**Figure 10**). A total of 58 proteins were co-regulated by ASF1B after the LC-MS were intersected (**Supplementary Figure 5**). Further study found POLE3, CKS1B, DHFR, ribosomal protein S29(RPS29), and transmembrane protein 230 (TMEM230) were affected by different biological background of cell lines (**Supplementary Table 1**). To confirm these results, we conducted Western blotting analyses of ASF1B-shRNA-H1975 and scrambled cells, revealing significant decreases in POLE3 expression consistent with these proteomic results (**Supplementary Figure 6**). ASFB1 expression was also associated with POLE3, CKS1B, and DHFR expression in most normal tissues and in many cancers including LUAD (R>0.4, p<0.05) (**Supplementary Figure 7**).

Immunofluorescent staining revealed ASF1B and POLE3 to localize to the nucleus, while CKS1B was present in the nucleus and cytoplasm (**Supplementary Figure 8**). We thus conducted an IP-MS experiment, which failed to reveal direct interaction between ASF1B and POLE3 or CKS1B (**Supplementary Figure 9**).

When we examined CKS1B expression in LUAD samples in the TCGA dataset, we found it was an independent predictor of poor LUAD patient prognosis and correlated with patient age, gender, T, N, M, and clinical stage (**Supplementary Figure 10**). POLE3 was unrelated to LUAD patient prognosis or clinicopathological parameters in LUAD. Therefore, we detected the mRNA expression of CKS1B in stable knockdown ASF1B cells and scramble cells. ASF1B knockdown reduced CKS1B mRNA expression, indicating ASF1B regulate CKS1B independent of post-transcriptional regulation (**Supplementary Figure 11**).

## Discussion

Herein, we examined the expression and prognostic relevance of ASF1B across many cancer types. In a TCGA analysis, we observed TCGA upregulation in 25 cancers other than LAML relative to corresponding normal tissue samples. Oncomine results were largely consistent with the results of these analyses. Many genes play different roles in different cancers (58–60), thus explaining the variable prognostic significance of ASF1B observed among cancer types and subtypes. Such tumor heterogeneity is a significant barrier to reliable tumor treatment (61–63). The onset and progression of cancer can be profoundly impacted by genetic and epigenetic changes, MSI, and TMB, and many of these mechanisms were correlated with ASF1B expression levels in different cancers in the present analysis.

In enrichment analyses, we found ASF1B was primarily associated with immune-, proliferation-, and autophagy-related pathways, some of which were enriched in both normal tissues and cancers although the associated genes differed. As such, we hypothesized that ASF1B may regulate immune cell infiltration by influencing genes in immune-related pathways.

We observed a close relationship between ASF1B and proliferation-related pathways including DNA replication and the cell cycle in LUAD. Our experimental results further confirmed that knocking down ASF1B impaired proliferation, altered cell cycle progression, and induced cell apoptosis in LUAD cells. In contrast, no impact of ASF1B overexpression was observed, possible because A549 and H1299 cells grow rapidly, and thus ASF1B overexpression may not further enhance their proliferation. The mechanisms whereby ASF1B can shape tumorigenesis remain poorly understood. Herein, we determined that in LUAD cells, ASF1B can indirectly regulate CKS1B, POLE3, and DHFR expression, and we found it positively correlated with the expression of these genes in most tumor and normal tissue samples. This indicates that ASF1B regulates cancer progression through these signaling pathways.

Notably, CKS1B is a CKS family protein that regulates cell cycle progression, growth, apoptosis, invasion metastasis, and chemical resistance in a range of cancer types (64–72). Wang et al. found that overexpression of CKS1B achieved in lung cancer cells through lentiviral infection enhanced drug resistance by inhibiting cisplatin (CDDP)- and doxorubicin (DOX)-induced apoptosis, supporting the critical role of CKS1B in lung cancer progression (73). A study has shown that CKS1B overexpression promoted drug resistance in myeloma. Moreover, research has demonstrated that CKS1B induces resistance to ubiquitin-like protein synthesis inhibitors such as bortezomib by inhibiting expression of the S-Phase Kinase Associated Protein 2/KIT Ligand (SCF/SKP2) substrate p21 (74, 75). DHFR is a ubiquitous enzyme and exists in a wide range of organisms (76). DHFR, a key enzyme in folate metabolism, converts dihydrofolate into tetrahydrofolate. It is well known that Pemetrexed and Methotrexate inhibits DHFR in the folate pathway, which is essential for the rapid cellular division and proliferation of cancer cells (77). Hence, the inhibition of DHFR can limit the growth and proliferation of cells. POLE3 is known subunits of DNA polymerase epsilon and more recently has been shown to form a newly identified histone H3-H4 chaperone complex that participates in the maintenance of chromatin integrity during DNA replication (78). Su et al. observed that POLE3-deficient cells displayed enhanced sensitivity to a Poly (ADP-Ribose) Polymerase (PA RP) inhibitor, an ATR inhibitor, and camptothecin (79, 80). Interestingly, above data demonstrated that targeting ASF1B may be an important method for cancer treatment. However, there are some limits. The detailed molecular mechanisms underlying the regulation of those key proteins by ASF1B need further explored in LUAD and other cancers. Nude mouse tumor formation experiment is also performed. Underlying mechanisms of immune infiltrate ion signaling pathways remain unclear, while function annotations and enrichment analysis of ASF1B are investigated.

In summary, we herein outlined the critical role played by ASF1B in LUAD cells, providing novel insight into its role as a regulator of cellular proliferation, cell cycle progression, and apoptotic induction. These data provide a more general framework for future studies of ASF1B in other cancer types and indicate that this protein may represent a viable therapeutic target in LUAD and other cancer types in the future.

## Data Availability Statement

The original contributions presented in the study are included in the article/**Supplementary Material**. Further inquiries can be directed to the corresponding author.

## Author Contributions

WC: also performs experimental operations. WZ: Analyzing data and writing manuscript. ZG, MG, and NL: Load raw data. GW: Concept and revised the manuscript. FJ: analyzing experiment data. All authors contributed to the article and approved the submitted version.

## Funding

This study was funded by Wu Jieping Medical Foundation (320.6750.19 089-55) and by Tianjin Health Science and Technology Project (ZC20125).

## Conflict of Interest

The authors declare that the research was conducted in the absence of any commercial or financial relationships that could be construed as a potential conflict of interest.

## Publisher’s Note

All claims expressed in this article are solely those of the authors and do not necessarily represent those of their affiliated organizations, or those of the publisher, the editors and the reviewers. Any product that may be evaluated in this article, or claim that may be made by its manufacturer, is not guaranteed or endorsed by the publisher.

## Supplementary Material

Supplementary Figure 1 CPG site correlated with prognosis of different cancers.

Supplementary Figure 2 ASF1B mRNA expression in different immune subtypes in most cancers via TISIDB.

Supplementary Figure 3 The mRNA expression of ASF1B in four cell lines were analyzed by using CCLE database.

Supplementary Figure 4 SiRNA interference sequences were screened in the protein level.

Supplementary Figure 5 The density levels were quantified and represented as a bar graph.

Supplementary Figure 6 Western blot analysis of POLE3 in H1975 cells treated with knockdown-ASF1B and untreated controls cells. The density levels were quantified and represented as a bar graph.

Supplementary Figure 7 Association ASF1B with CKS1B, POLE3 and DHFR in pan-cancers and normal tissues.

Supplementary Figure 8 ASF1B is co-localized with CKS1B and POLE3 in cell nucleus.

Supplementary Figure 9 Immunoprecipitation Mass spectrometry of A549 cell line induced by overexpressed of ASF1B. Differentially expressed proteins in stable ASF1B-OE cells compared to negative control. (Volcano plot) Red presents up-regulated proteins, blue represents down-regulated proteins, and black presents no significantly differentially expressed proteins. (Heatmap) The expression patterns of these differentially expressed proteins can distinguish between stable ASF1B-OE cell line and negative control.

Supplementary Figure 10 CKS1B correlated with prognosis and clinicopathology.

Supplementary Figure 11 Real-time PCR analysis of mRNA expression of ASF1B in H1975 Scramble cell lines compared to H1975 SH ASF1B cell lines.

Supplementary Table 1 The results of LC-MS from four group cell lines were intersected(Log FC).
