# Supplementary material for: ASF1B Promotes Oncogenesis in Lung Adenocarcinoma and Other Cancer Types
Source: Front Oncol. 2021 Sep 9;11:731547. doi: 10.3389/fonc.2021.731547 (PMC8459715; doi:10.3389/fonc.2021.731547)

Survival Plot for the CpG cg25274248 in KIRP

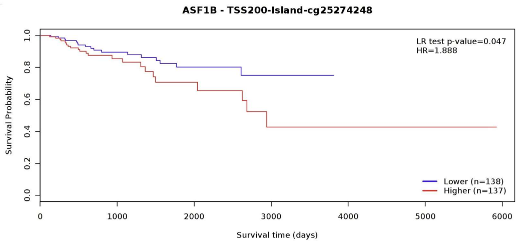

Survival Plot for the CpG cg26259181 in LGG

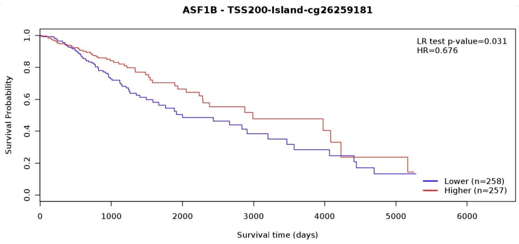

Survival Plot for the CpG cg25274248 in LIHC

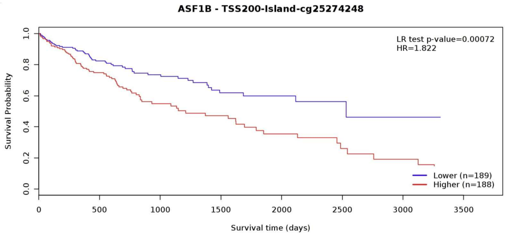

Survival Plot for the CpG cg26259181 in LUAD

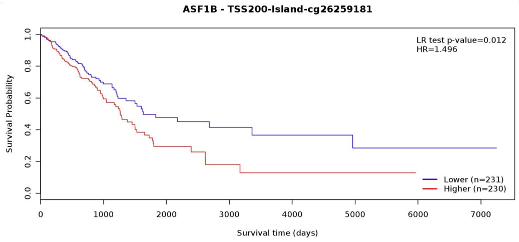

Survival Plot for the CpG cg06391548 in STAD

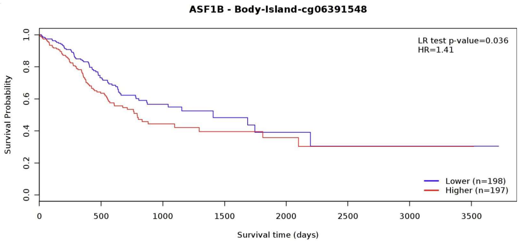

Supplement: Supplementary Figure 1 — CPG site correlated with prognosis of different cancers. [file DataSheet_1.pdf]
