# Supplementary figures and images for: ASF1B Promotes Oncogenesis in Lung Adenocarcinoma and Other Cancer Types
Source: Front Oncol. 2021 Sep 9;11:731547. doi: 10.3389/fonc.2021.731547 (PMC8459715; doi:10.3389/fonc.2021.731547)

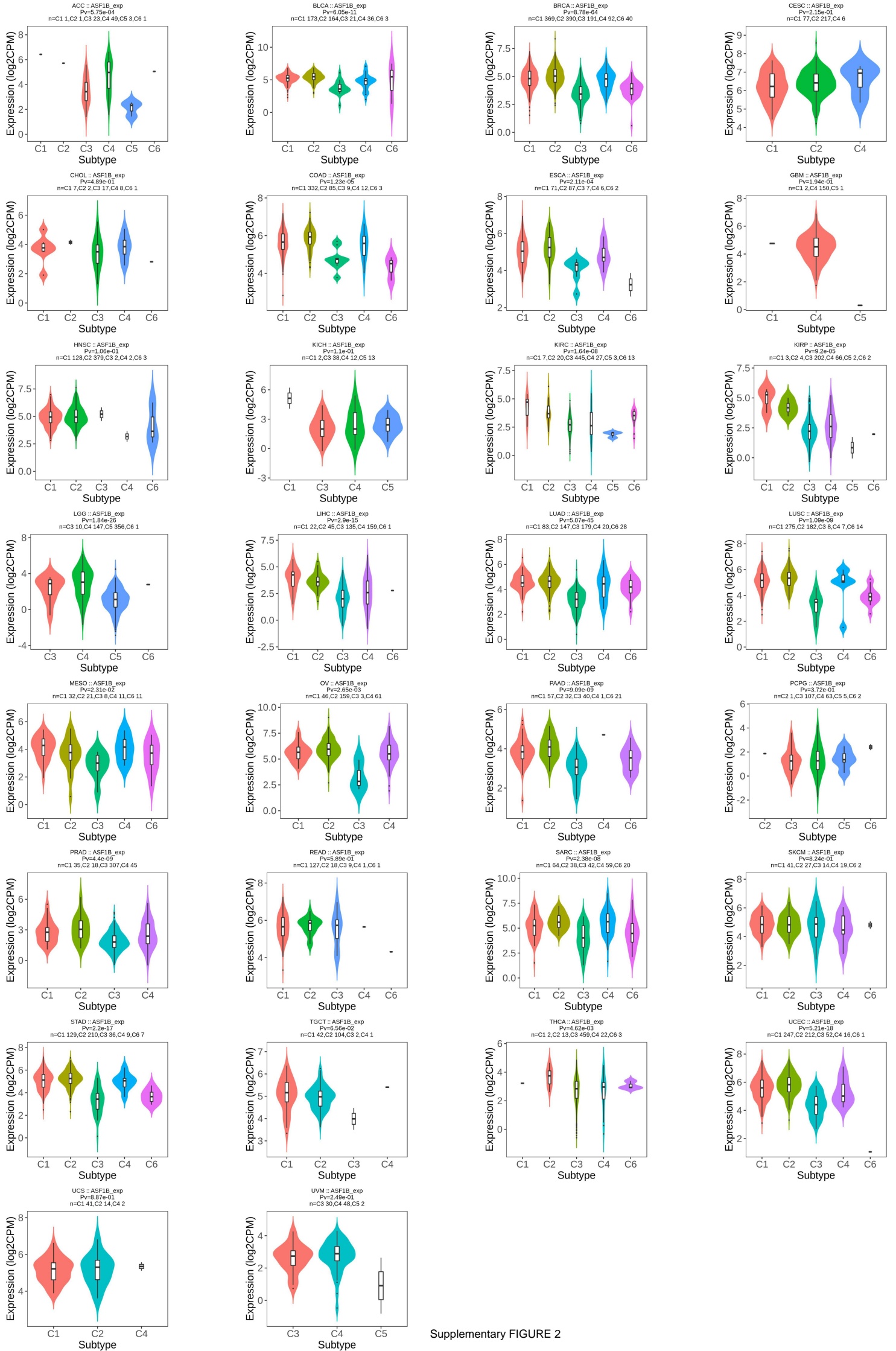

Supplementary FIGURE 2

Supplement: Supplementary Figure 2 — ASF1B mRNA expression in different immune subtypes in most cancers via TISIDB. [file DataSheet_2.pdf]

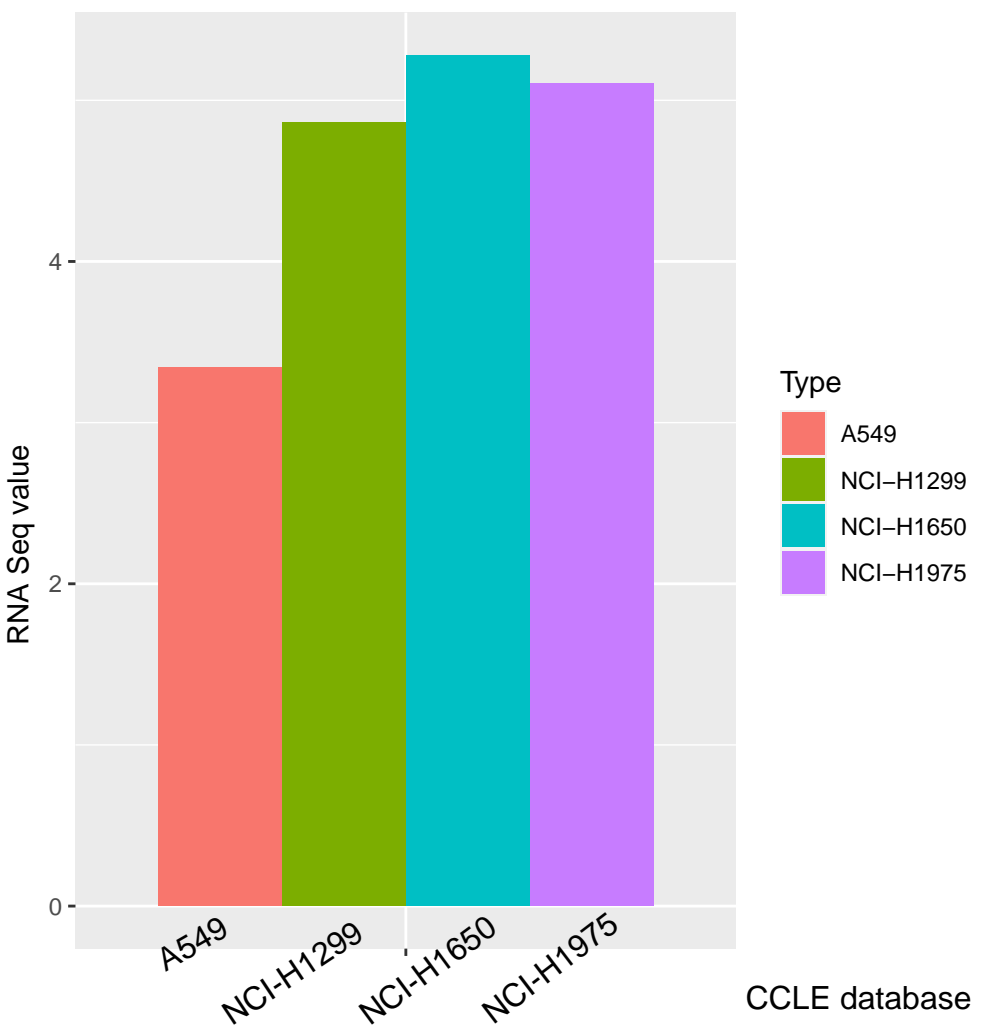

Supplement: Supplementary Figure 3 — The mRNA expression of ASF1B in four cell lines were analyzed by using CCLE database. [file DataSheet_3.pdf]

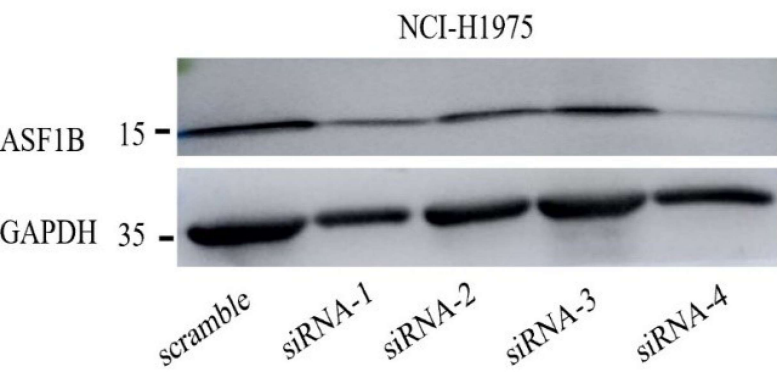

A

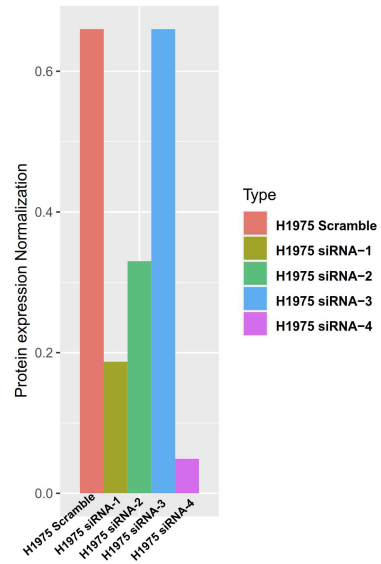

B

Supplement: Supplementary Figure 4 — SiRNA interference sequences were screened in the protein level. [file DataSheet_4.pdf]

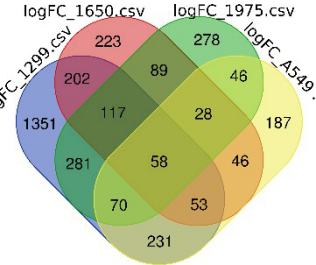

Supplement: Supplementary Figure 5 — The density levels were quantified and represented as a bar graph. [file DataSheet_5.pdf]

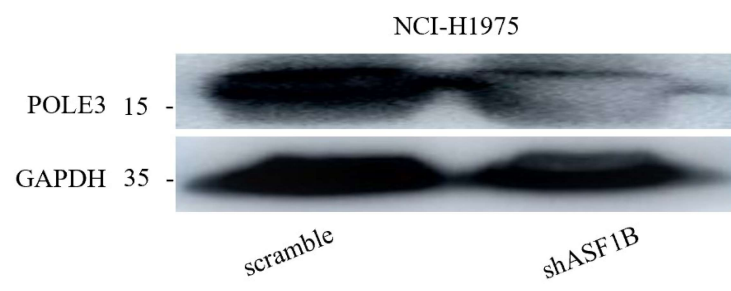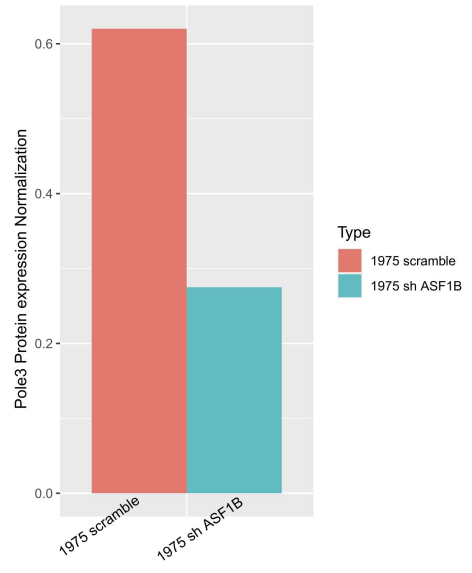

Supplement: Supplementary Figure 6 — Western blot analysis of POLE3 in H1975 cells treated with knockdown-ASF1B and untreated controls cells. The density levels were quantified and represented as a bar graph. [file DataSheet_6.pdf]

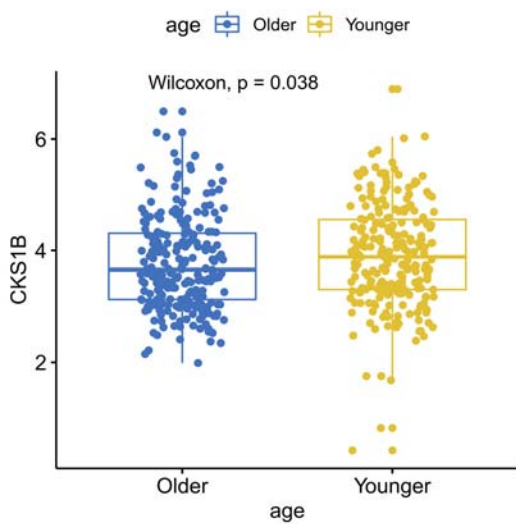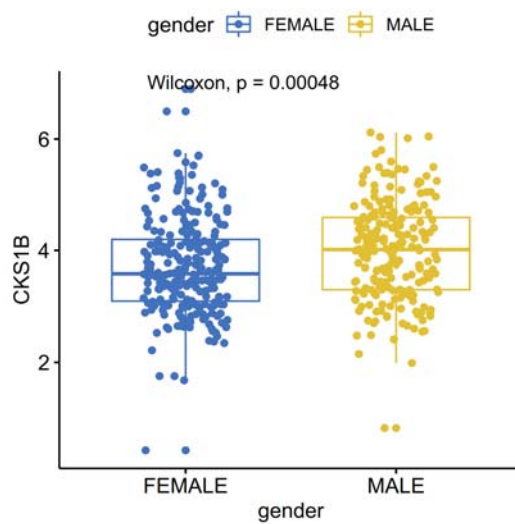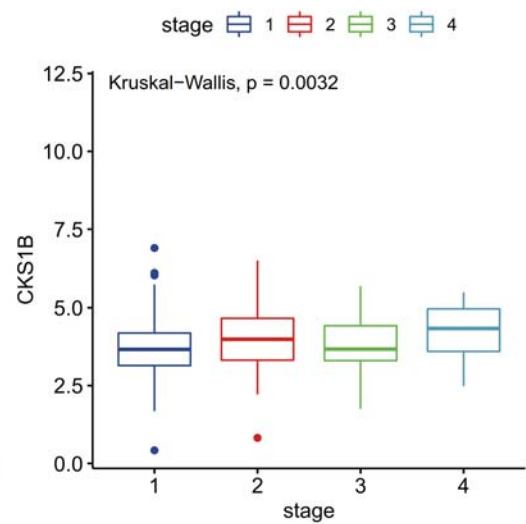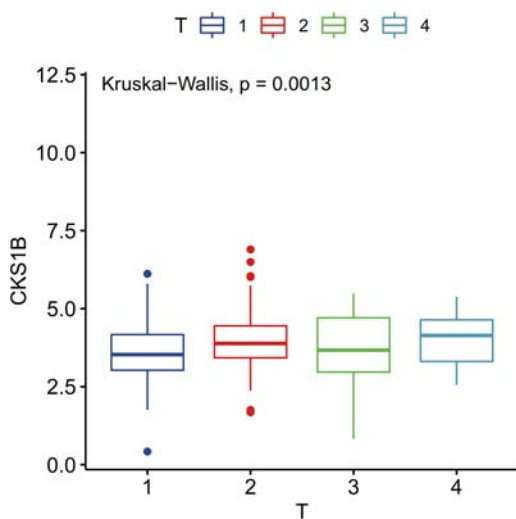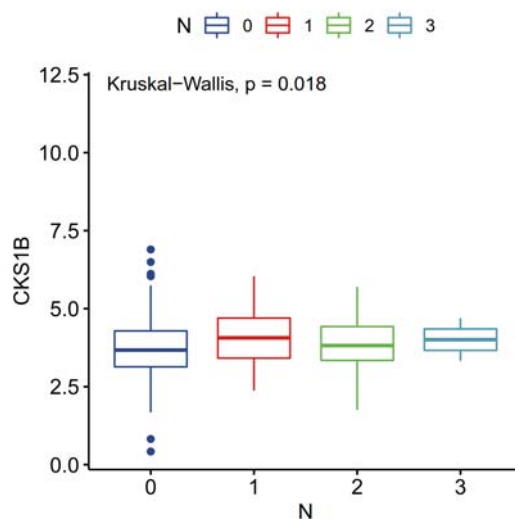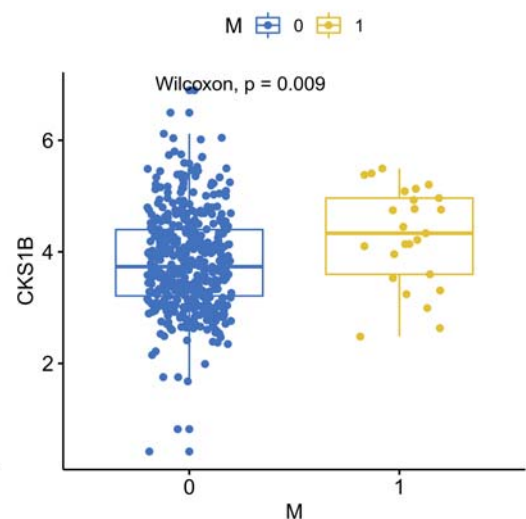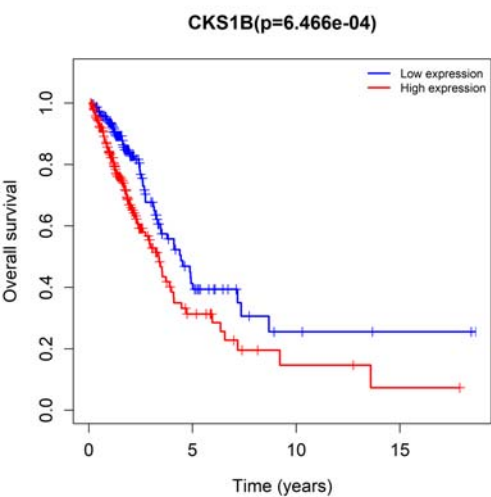

Supplement: Supplementary Figure 9 — Immunoprecipitation Mass spectrometry of A549 cell line induced by overexpressed of ASF1B. Differentially expressed proteins in stable ASF1B-OE cells compared to negative control. (Volcano plot) Red presents up-regulated proteins, blue represents down-regulated proteins, and black presents no significantly differentially expressed proteins. (Heatmap) The expression patterns of these differentially expressed proteins can distinguish between stable ASF1B-OE cell line and negative control. [file DataSheet_9.pdf]

Gene Expression

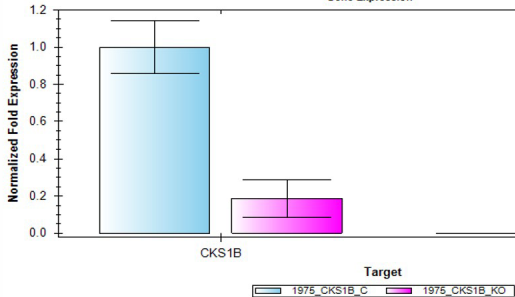

Melt Peak

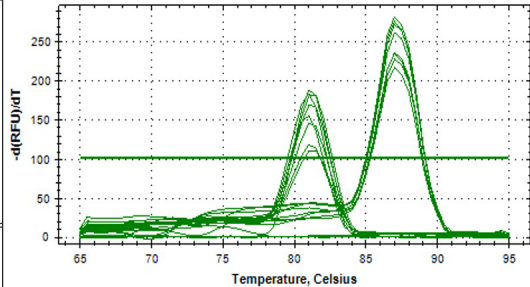

Supplement: Supplementary Figure 10 — CKS1B correlated with prognosis and clinicopathology. [file DataSheet_10.pdf]

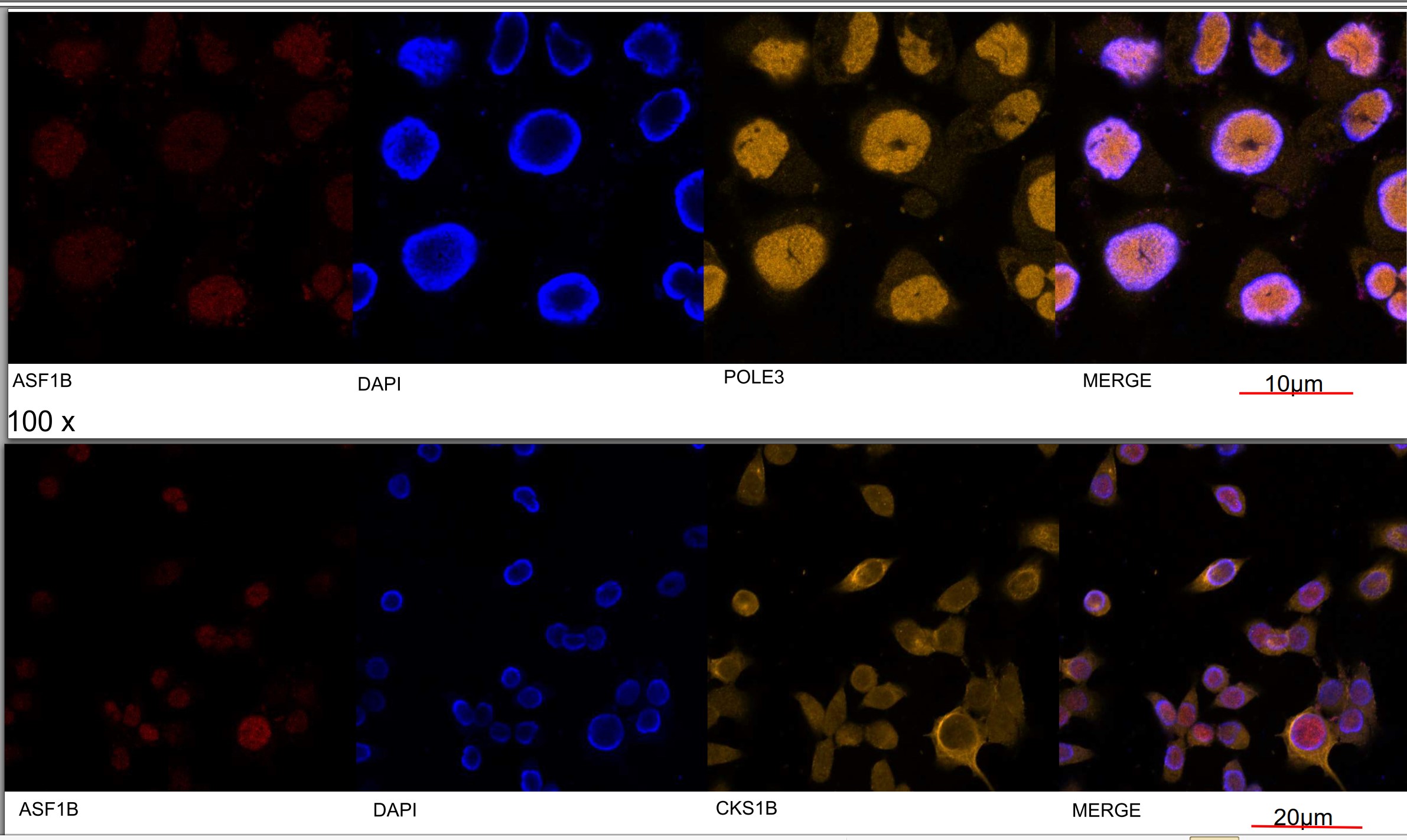

Supplement: Supplementary Figure 11 — Real-time PCR analysis of mRNA expression of ASF1B in H1975 Scramble cell lines compared to H1975 SH ASF1B cell lines. [file Image_1.jpeg]
