# Supplementary material for: ASF1B Promotes Oncogenesis in Lung Adenocarcinoma and Other Cancer Types
Source: Front Oncol. 2021 Sep 9;11:731547. doi: 10.3389/fonc.2021.731547 (PMC8459715; doi:10.3389/fonc.2021.731547)
Supplement: Supplementary Figure 7 — Association ASF1B with CKS1B, POLE3 and DHFR in pan-cancers and normal tissues. [file DataSheet_7.pdf]

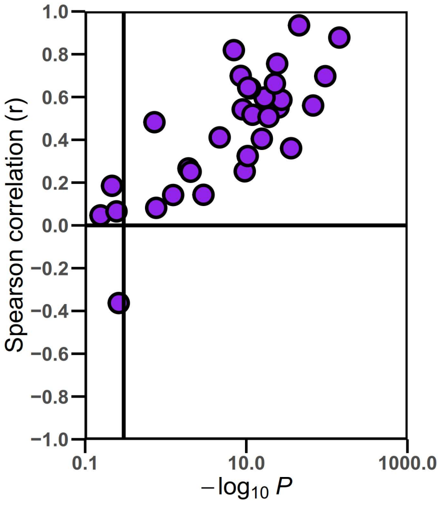

Correlation between ASF1B and CKS1B in GTEx

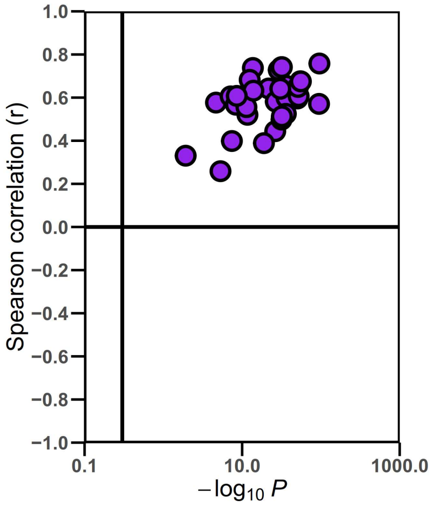

Correlation between ASF1B and CKS1B in pan-cancers

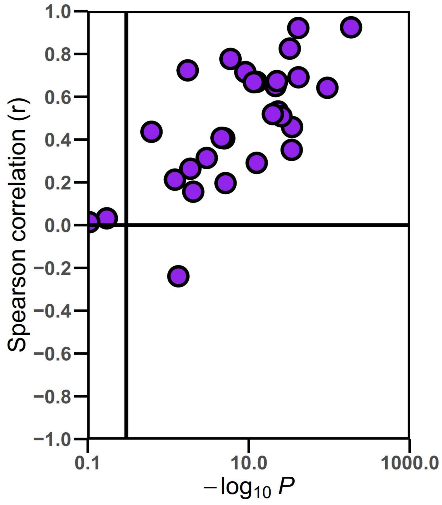

Correlation between ASF1B and DHFR in GTEx

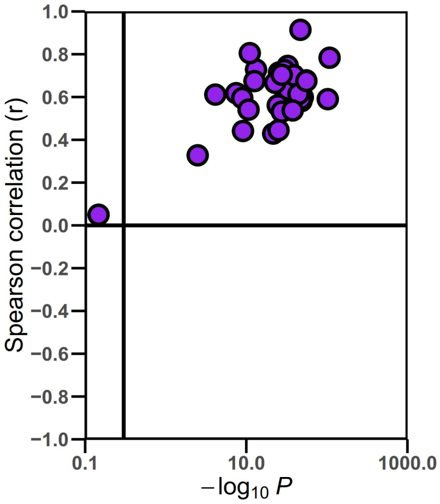

Correlation between ASF1B and DHFR in pan-cancers

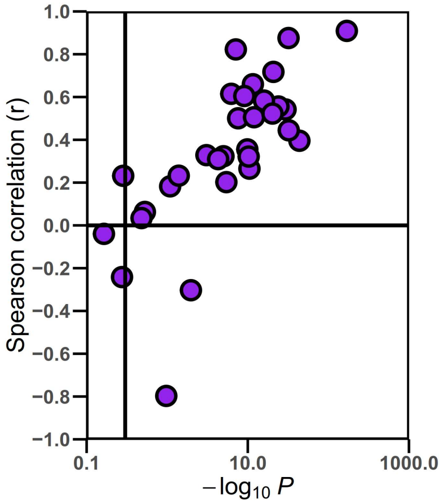

Correlation between ASF1B and POLE3 lin GTEx

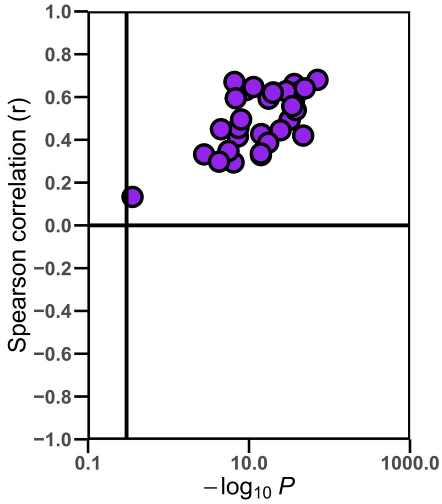

Correlation between ASF1B and POLE3 in pan-cancers

Supplementary Fig. 7
