# Supplementary material for: ASF1B Promotes Oncogenesis in Lung Adenocarcinoma and Other Cancer Types
Source: Front Oncol. 2021 Sep 9;11:731547. doi: 10.3389/fonc.2021.731547 (PMC8459715; doi:10.3389/fonc.2021.731547)
Supplement: Supplementary Table 1 — The results of LC-MS from four group cell lines were intersected(Log FC). [file Table_1.docx]

Supplementary Table 1

The results of LC-MS from four group cell lines were intersected（logFC）

| id | H1975 | H1650 | A549 | H1299 |
| --- | --- | --- | --- | --- |
| ATP5I | -1.0887 | -2.66697 | 0.681088 | -1.07561 |
| AVL9 | -0.6157 | 0.618728 | 0.887294 | 0.646266 |
| C19orf10 | 2.564961 | -0.8 | -1.09043 | -0.85812 |
| CBFB | -2.41831 | -0.74154 | -0.55486 | 0.739116 |
| CD151 | -1.34358 | 0.896247 | 1.628674 | 1.063854 |
| CD9 | 1.188566 | -1.33329 | 1.997051 | 2.204876 |
| CHMP2A | -1.17081 | -1.53075 | -0.68655 | -0.70289 |
| CHP1 | -0.80854 | -0.61911 | -0.68309 | 0.744838 |
| CKS1B | -1.62703 | -0.93669 | 0.511246 | 1.579579 |
| DCTN4 | -0.50887 | -1.58916 | -0.65338 | -1.29265 |
| DHFR | -1.14943 | -1.35133 | 0.968963 | 1.346774 |
| DYNLRB2 | 0.981102 | 0.842119 | -0.75351 | 1.822943 |
| FAHD1 | -0.82444 | 0.807562 | 0.96976 | -2.1248 |
| FN1 | 1.811726 | 0.582149 | 0.772926 | 2.458215 |
| GLS | -1.31546 | -1.07446 | 0.702861 | -0.78634 |
| GMFB | -1.18396 | 3.364591 | 1.79456 | 0.632497 |
| GNL1 | 1.363435 | -0.72717 | 1.667881 | 0.946347 |
| H1FX | -0.79251 | 0.68086 | -1.30962 | -2.07711 |
| HIST2H3A | -2.58489 | -2.44439 | 2.405741 | -3.89475 |
| HIST2H3C | -2.58489 | -2.44439 | 2.405741 | -3.89475 |
| HIST2H3D | -2.58489 | -2.44439 | 2.405741 | -3.89475 |
| HLA-B | 1.142946 | 0.670553 | 1.112484 | 0.576091 |
| HLA-C | 0.708361 | 0.575238 | 0.810729 | 0.745387 |
| ISOC1 | -0.50385 | -1.03738 | -0.52607 | 0.714175 |
| KRT24 | 1.599733 | -1.48208 | 0.999497 | -0.80045 |
| KRT27 | 1.966842 | -1.37389 | 1.365151 | -0.6085 |
| LAMP1 | -0.65536 | 0.572101 | -1.68217 | -1.73096 |
| MAGT1 | 0.518684 | 0.78428 | 0.548848 | -0.70752 |
| MRPL13 | -0.96798 | 0.690152 | 1.295582 | 0.522195 |
| MRPL18 | -2.09267 | 1.664668 | 1.108621 | -1.64653 |
| MRPL22 | -1.46277 | -1.1923 | 0.865883 | -0.93063 |
| MRPL3 | -1.22795 | 0.520447 | -0.79027 | -1.60228 |
| NDUFA13 | -2.0575 | 3.390865 | 1.193104 | -1.76824 |
| NDUFA5 | -0.53271 | -0.94786 | 0.819672 | -0.54472 |
| NEDD8 | -1.21512 | -1.46483 | -1.50116 | -3.96913 |
| NFU1 | 0.575673 | -0.72691 | 0.537403 | -1.75995 |
| OSTF1 | -1.11575 | 1.075094 | -1.85177 | 2.051986 |
| PARN | -1.43541 | -0.93658 | -0.80792 | -2.43618 |
| PGRMC1 | 0.609589 | 0.700978 | 0.746114 | 0.873634 |
| POLE3 | -2.32483 | -1.12966 | 1.119979 | 1.436679 |
| RBMXL2 | -0.58879 | 0.856939 | -0.60431 | -2.22976 |
| RNF114 | 0.990464 | 0.64142 | 0.571269 | 0.652406 |
| RPS27 | -0.54245 | -2.01811 | -3.48878 | 1.393597 |
| RPS29 | 1.74441 | 1.732043 | -1.51395 | -4.44499 |
| RTN3 | -0.93115 | -0.74193 | -1.0234 | -1.24513 |
| SEC61A1 | -0.70597 | 0.503383 | -0.73646 | -0.75938 |
| SF3B14 | -0.5544 | -1.0655 | 1.215305 | -1.18631 |
| SFXN3 | 0.520091 | 0.837828 | 1.206772 | 0.742107 |
| SLC25A10 | -3.21485 | 1.549763 | -0.74982 | -1.10167 |
| SNRPE | -0.59413 | -1.89509 | 0.627433 | -0.84038 |
| SPATS2L | -0.83197 | 0.528302 | 0.637998 | 1.41931 |
| TMEM230 | 0.590737 | 2.02123 | -0.52456 | -0.51007 |
| TOMM5 | 0.700133 | -2.39023 | 1.288822 | 0.590645 |
| TRUB1 | -1.10337 | -1.07856 | 0.871275 | -0.69573 |
| UBXN4 | -0.75652 | -0.50908 | -0.96389 | -0.56965 |
| UQCR10 | -1.69451 | -2.01099 | 1.27958 | -3.13698 |
| ZC3H11A | -1.57838 | -1.21004 | -0.94232 | -0.58758 |
