# Supplementary material for: ASF1B Promotes Oncogenesis in Lung Adenocarcinoma and Other Cancer Types
Source: Front Oncol. 2021 Sep 9;11:731547. doi: 10.3389/fonc.2021.731547 (PMC8459715; doi:10.3389/fonc.2021.731547)
Supplement: Supplementary Figure 8 — ASF1B is co-localized with CKS1B and POLE3 in cell nucleus. [file DataSheet_8.pdf]

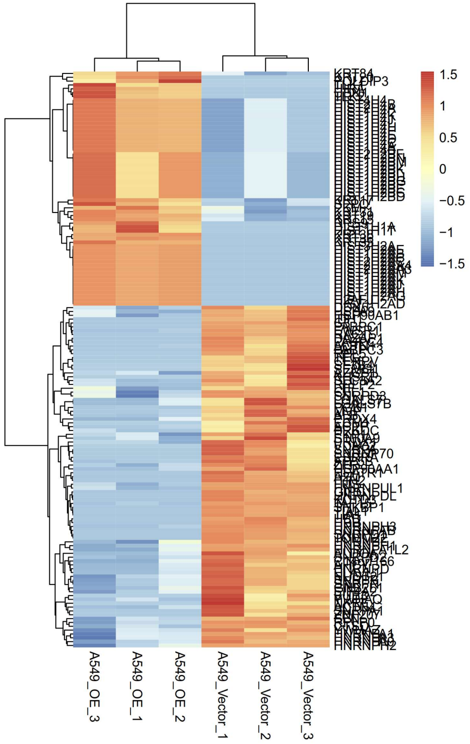
1.5
1
0.5
0
−0.5
−1
−1.5
A549_OE_3
A549_OE_1
A549_OE_2
A549_Vector_1
A549_Vector_2
A549_Vector_3

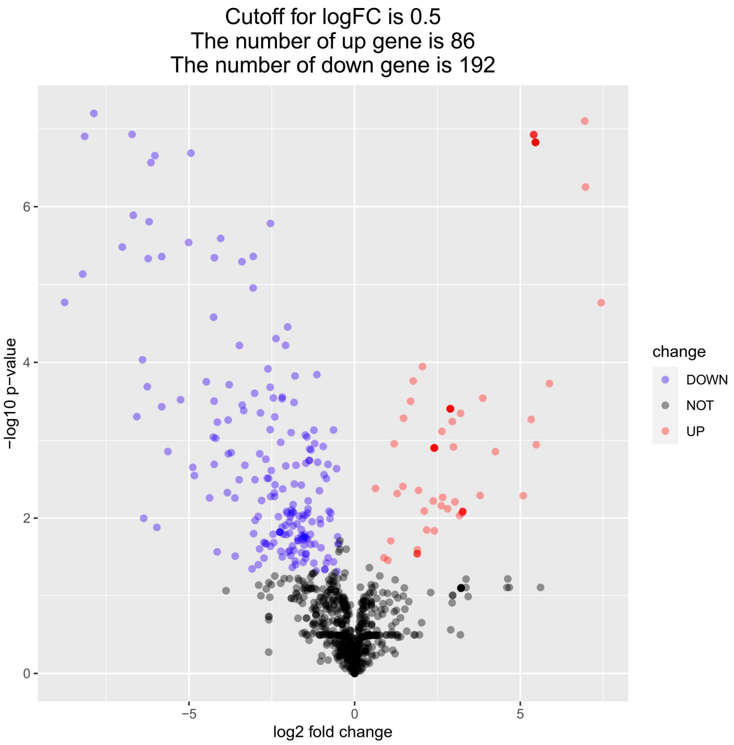
Cutoff for logFC is 0.5
The number of up gene is 86
The number of down gene is 192
−log10 p−value
log2 fold change
change
DOWN
NOT
UP
6
4
2
0
−5
0
5
